# Characterization of ocular adverse events associated with crizotinib: real-world insights from the two global pharmacovigilance databases of FAERS and VigiBase

**DOI:** 10.3389/fonc.2025.1735200

**Published:** 2025-12-12

**Authors:** Shuai Dong, Zhi-chao Jiang, Li Gao, Rong Zhang, Yan Liu

**Affiliations:** The Third People’s Hospital of Dalian, Liaoning, China

**Keywords:** crizotinib, ocular adverse events, FAERS, VigiBase, disproportionality analysis

## Abstract

To evaluate the risk of ocular adverse events (AEs) associated with crizotinib using real-world data from FAERS and VigiBase, and to characterize signal patterns through disproportionality and time-to-onset (TTO) analyses. Reports from FAERS and VigiBase (2011–2025) were analyzed. Disproportionality was assessed using reporting odds ratio (ROR) at both system organ class (SOC) and preferred term (PT) levels. TTO analysis was estimated based on FAERS data. Crizotinib was consistently associated with ocular AEs across both databases (FAERS ROR = 3.46, 95% confidence interval [CI]: 3.30–3.63; VigiBase ROR = 3.34, 95% CI: 3.17–3.51). Frequent PTs included visual impairment, blurred vision, and photopsia. High RORs were also observed for common events such as photopsia (FAERS: ROR = 42.5; VigiBase: ROR = 49.42) and visual brightness (FAERS: ROR = 31.67; VigiBase: ROR = 214.25). The median TTO was 14 days, suggesting early onset during treatment. Crizotinib is associated with a distinct profile of ocular AEs, typically mild and early in onset. The detection of rare but strongly associated PTs underscores the need for routine ophthalmologic monitoring. These findings are biologically plausible, as crizotinib inhibits the MET and ROS1 signaling pathways, both of which are expressed in retinal tissue, thereby supporting the need for enhanced clinical vigilance in patients receiving crizotinib.

## 1 Introduction

Non-small-cell lung cancer (NSCLC) remains the leading cause of cancer-related mortality worldwide, accounting for approximately 85% of all lung cancer cases (1, 2). Rearrangements of the anaplastic lymphoma kinase (ALK) gene occur in 3–7% of NSCLC cases and confer marked sensitivity to ALK tyrosine kinase inhibitors (TKIs) (3, 4). The identification of ALK rearrangements has enabled the development of molecularly targeted therapies, substantially improving outcomes for patients with advanced ALK-positive NSCLC (5, 6). Crizotinib, a first-generation ALK TKI approved in 2011, established a new standard of care for this molecular subtype. Compared to chemotherapy, crizotinib significantly prolongs median progression-free survival (7.7 *vs.* 3.0 months) and achieves a higher objective response rate (70.0%) with a median duration of response of 27.8 months (7–9).

Crizotinib has demonstrated efficacy in treating NSCLC patients with ROS1 rearrangements and c-MET mutations, thereby broadening its clinical indications (5, 6). However, its use is associated with a range of adverse events (AEs), including elevated transaminases, visual disturbances, bradycardia, QT interval prolongation, gastrointestinal symptoms (nausea, vomiting, diarrhea, constipation), and interstitial pneumonia (8, 10). Evidence from observational cohort studies on ocular toxicity remains inconsistent, with reporting frequency of vision disorders as low as 12.5 per 1000 person-years in Europe and 47.8 per 1000 person-years in the United States (11). Such underestimation is likely due to the mild and transient nature of most visual AEs, which are frequently underreported. Accordingly, a large-scale real-world investigation is warranted to comprehensively evaluate the ocular safety profile of crizotinib.

The FDA Adverse Event Reporting System (FAERS) and VigiBase are two major spontaneous reporting databases that provide critical real-world evidence for post-marketing pharmacovigilance (12). FAERS, maintained by the U.S. Food and Drug Administration, is extensively utilized to detect safety signals related to AEs associated with approved drugs and biologics (13). In parallel, VigiBase—developed and managed by the Uppsala Monitoring Centre on behalf of the World Health Organization—serves as the global repository for suspected adverse drug reactions, facilitating early detection of emerging safety concerns across diverse populations and therapeutic contexts (14).

Given the increasing clinical use of crizotinib, a comprehensive understanding of its adverse effect profile—particularly ocular toxicity—is essential for optimizing patient safety and informing therapeutic strategies. This study utilizes standardized data from FAERS and VigiBase to evaluate the risk of crizotinib-associated ocular AEs, aiming to refine its safety profile and provide evidence-based guidance for healthcare professionals and policy decision-makers in the management of these events.

## 2 Materials and methods

### 2.1 Data source

The FAERS (https://fis.fda.gov/extensions/FPD-QDE-FAERS/FPD-QDE-FAERS.html) and VigiBase (https://www.vigiaccess.org/) are publicly accessible pharmacovigilance databases that aggregate global reports of AEs, medication errors, and product quality complaints submitted by healthcare professionals and consumers. These complementary systems serve as vital tools for post-marketing drug safety surveillance and enable comprehensive assessments of medication-associated risks. This study involved human participants and complied with relevant laws and institutional policies. Accordingly, ethical approval was not required. In line with national and institutional regulations, written informed consent from participants or their legal guardians/relatives was not mandatory for participation.

**Figure 1:**
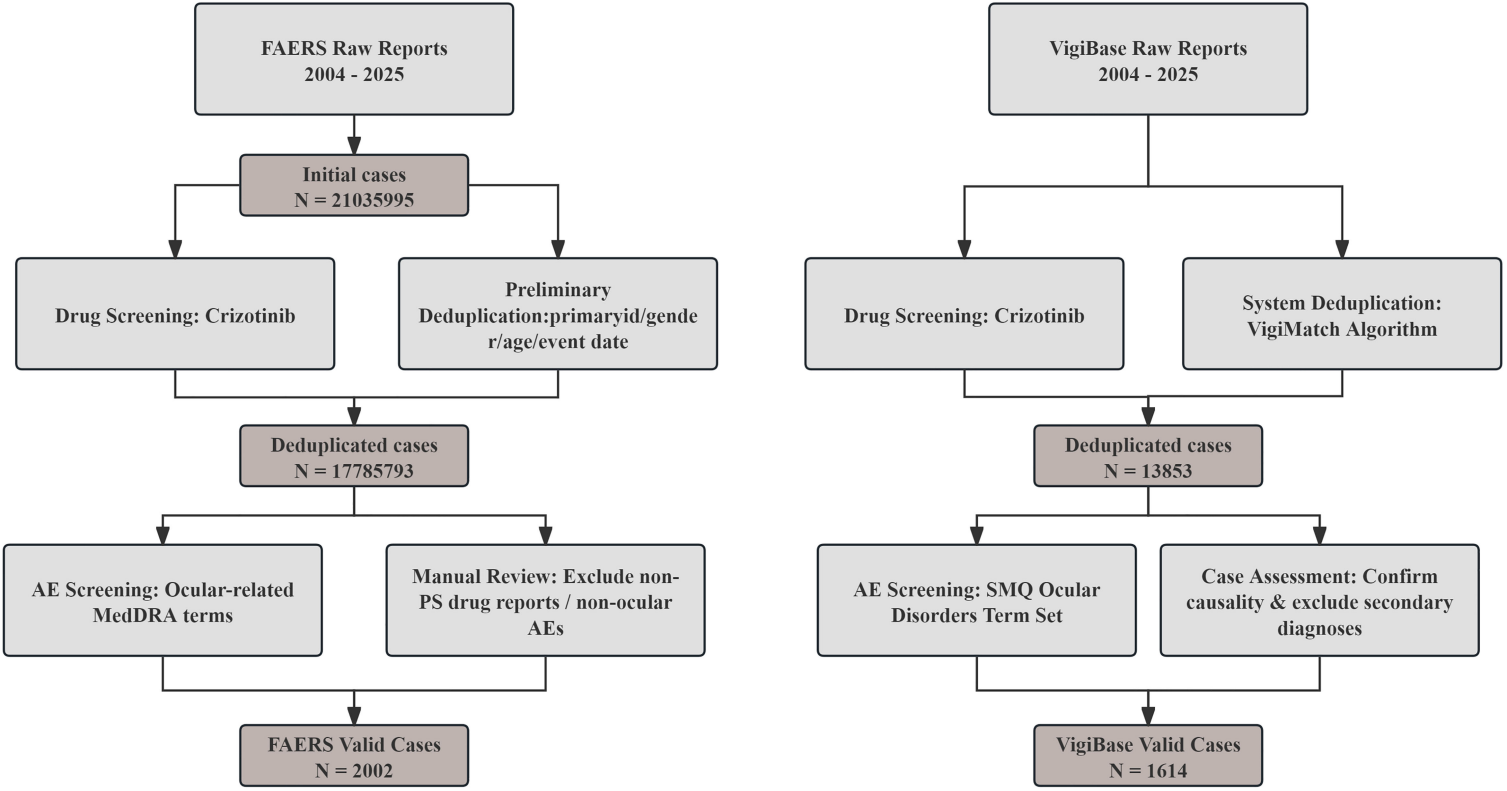
Flow diagram for the selection of ocular AEs with crizotinib from the FAERS and VigiBase databases.

For this study, AE reports related to crizotinib were extracted from both the FAERS and VigiBase databases. The retrieval period extended from database inception to the first quarter of 2025 for FAERS, and through 2025 for VigiBase. Extracted data included patient demographics (age and gender) and AE distributions categorized by system organ class (SOC). To ensure data accuracy and reliability, a systematic deduplication procedure was implemented to eliminate duplicate entries and retain only complete and relevant records for subsequent analysis. The overall data processing workflow is depicted in **Figure 1**.

### 2.2 AE description methodology

Due to differences in data granularity between databases, the frequency and distribution of AEs in FAERS and VigiBase were analyzed using the Standardised MedDRA Query (SMQ) framework, specifically employing the SOC hierarchy from the Medical Dictionary for Regulatory Activities (MedDRA) (9). MedDRA classifies AEs into broad SOC categories, which are further subdivided into specific Preferred Terms (PTs), allowing for consistent and standardized classification of reported events across pharmacovigilance systems.

### 2.3 Data extraction and processing

Data extraction was performed using the OpenVigil 2.1 tool for both the FAERS and VigiBase databases. Searches were conducted using the generic name “crizotinib,” with filters applied to retain only records in which crizotinib was designated as the primary suspected drug. To enhance the validity of the analysis, duplicate entries and reports related solely to the drug’s indications were excluded. AEs were coded using PTs from the most recent version of the MedDRA and subsequently classified according to their corresponding SOC categories (15). Furthermore, the time to onset of ocular AEs was assessed, defined as the interval between the initiation of crizotinib therapy and the occurrence of the event. Only cases with a reported onset time greater than zero days were included in the final analysis (16).

### 2.4 Signal mining methodology

Disproportionality analysis, a widely accepted method in post-marketing pharmacovigilance, served as the primary approach for signal detection in this study. Four standard measures were employed: Reporting Odds Ratio (ROR), Proportional Reporting Ratio (PRR), Empirical Bayes Geometric Mean (EBGM), and Bayesian Confidence Propagation Neural Network (BCPNN). These complementary algorithms were applied to identify potential safety signals of ocular AEs associated with crizotinib. Data extraction and statistical analyses were independently performed by two investigators using IBM^®^ SPSS^®^ Statistics (version 27.0) and R software (version 4.3) to ensure analytic robustness and reproducibility.

## 3 Results

### 3.1 Descriptive analysis

In this study, adverse events (AEs) associated with crizotinib were retrieved from both the FAERS and VigiBase databases, and ocular AEs were analyzed as a predefined subset. A total of 24,443 crizotinib-related AE reports were identified across all system organ classes (SOCs), comprising 10,590 reports from FAERS and 13,853 from VigiBase (**Table 1**). Among these, 2,002 FAERS reports and 1,614 VigiBase reports were classified under the “Eye disorders” SOC and were used for ocular signal detection (**Table 2**). In FAERS, 5,209 reports (49.19%) originated from female patients and 4,258 (40.21%) from male patients. Similarly, in VigiBase, 6,874 reports (49.62%) were submitted for female patients and 5,624 (40.60%) for male patients, suggesting a slightly higher reporting frequency of ocular AEs among females in both datasets. In terms of body weight distribution (available in FAERS only), the majority of patients weighed between 50 and 100 kg (n = 1,928; 18.21%), while smaller proportions weighed less than 50 kg (n = 432) or more than 100 kg (n = 152).

**Table 1:** Reported characteristics of ocular adverse events associated with crizotinib.

Characteristics	FAERS (n=10590)	VigiBase (n=13853)
Gender, n (%)		
Female	5209	6874
Male	4258	5624
Unknown	1123	1355
Weight (kg), n (%)		
<50 kg	432	
>100 kg	152	
50~100 kg	1928	
Missing	8078	
Age (years), n (%)		
<18	34	283
>18, <64	1264	5846
≥65, <74	483	2566
≥ 74 years	731	1678
Missing	8078	3480
Reported countries, n (%)		
US	5595	6405
Non-US	4995	7448
Occupation of reporters, n (%)		
Consumer (CN)	3576	
Health Professional (HP)	376	
Physician (MD)	3794	
Pharmacist (PH)	1154	
Other health-professional (OT)	1580	
Unknown	110	
Outcomes, n (%)		
Death (DE)	3359	
Disability (DS)	36	
Hospitalization (HO)	1677	
Life-Threatening (LT)	155	
Other serious (Important medical event) (OT)	1901	
Required intervention to prevent permanent impairment/damage (RI)	4	
Unknown	3458	

N, number of total ocular event reports.

**Table 2:** Signal detection of crizotinib-associated eye disorder adverse events from two databases.

Crizotinib	The report number	ROR (95%CI)	PRR (χ^2^)	EBGM (EBGM05)	IC (IC025)
FAERS	2002	3.46 (3.3– 3.63)	3.3 (3100.26)	3.29 (3.17)	1.72 (0.05)
VigiBase	1614	3.34 (3.17– 3.51)	3.28 (2483.46)	3.2 (3.06)	1.68 (0.01)

PRR, the proportional reporting ratio; ROR, the reporting odds ratio; IC, the information component; EBGM, the empirical Bayes geometric mean; CI, confidence interval; 95%CI, two-sided for ROR; **χ**^2^, chi-squared; IC025 and EBGM05 lower one-sided for IC, and EBGM.

**Figure 2:**
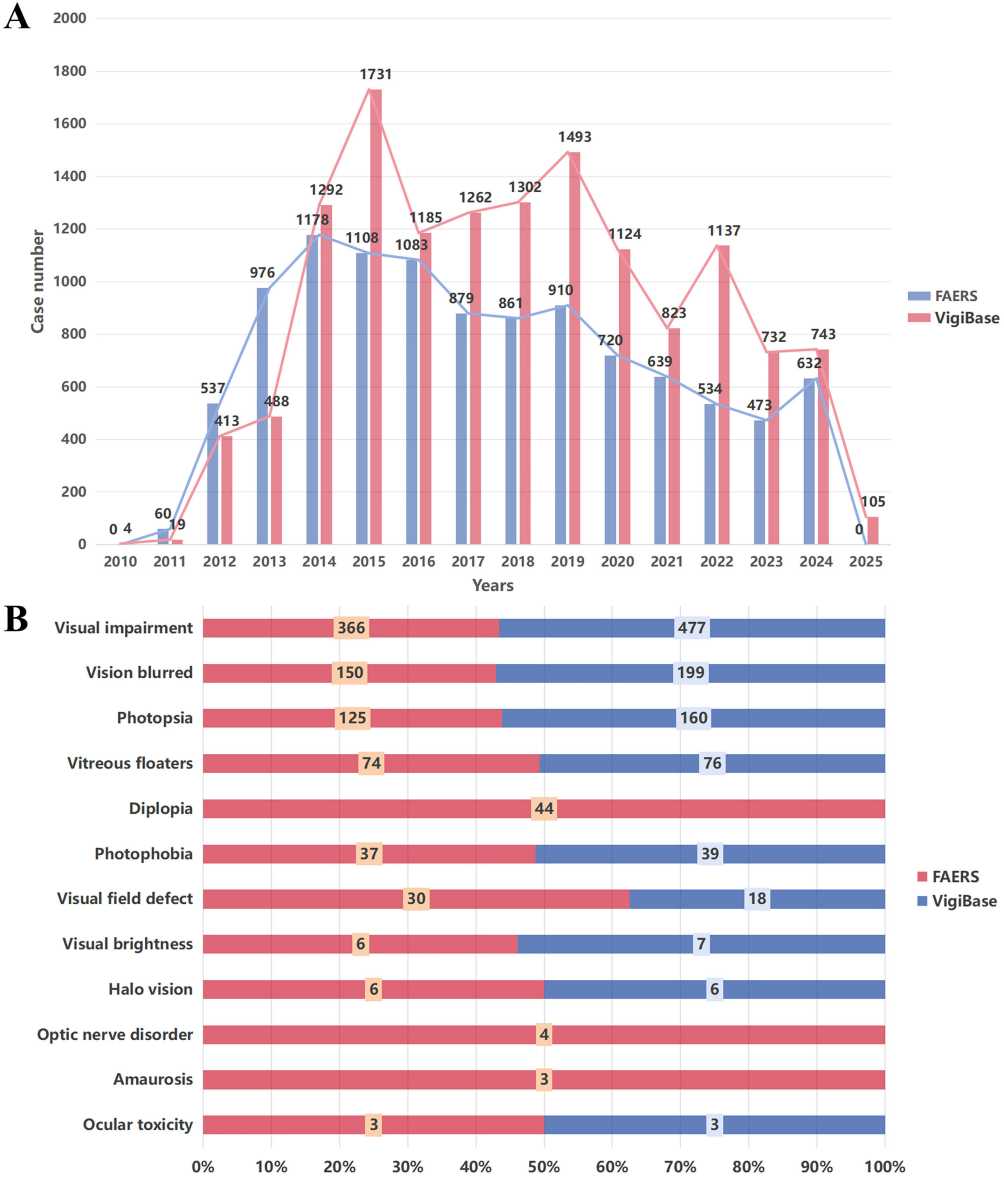
**(A)** The annal reporting frequency from 2010 to 2025 was calculated by dividing the number of reported cases associated with crizotinib. **(B)** Statistical analysis of crizotinib related ocular adverse events occurrences in FAERS and VigiBase databases spanning 2010–2025.

In terms of age distribution, the majority of crizotinib-related ocular AE reports in FAERS involved patients aged 18 to 64 years (n = 1,264; 11.94%), followed by those aged ≥74 years (n = 731; 6.90%) and 65 to 74 years (n = 483; 4.56%). In VigiBase, a similar pattern was observed, with the highest proportion of reports from patients aged 18 to 64 years (n = 5,846; 42.20%), followed by 65 to 74 years (n = 2,566; 18.52%) and ≥74 years (n = 1,678; 12.11%). Geographically, in FAERS, most reports originated from the U.S. (n = 5,595; 52.83%), while non-U.S. sources accounted for 4,995 reports (47.17%). In contrast, VigiBase contained a higher proportion of non-U.S. reports (n = 7,448; 53.76%) compared to those from the United States (n = 6,405; 46.24%). Temporal distribution analysis revealed that the peak reporting years were 2014 for FAERS (1,178 cases) and 2015 for VigiBase (1,731 cases), as shown in **Figure 2A**.

Regarding reporter occupation in the FAERS database, physicians submitted the largest proportion of crizotinib-related ocular AE reports (n = 3,794; 35.83%), followed by consumers (n = 3,576; 33.77%), other health professionals (n = 1,580; 14.92%), and pharmacists (n = 1,154; 10.90%). In terms of reported outcomes, hospitalization was documented in 1,677 cases (15.84%), while important medical events were noted in 1,901 reports (17.95%). Notably, death was reported in 3,359 cases (31.72%), reflecting the potential severity of AEs associated with crizotinib exposure.

### 3.2 Ocular AEs associated with crizotinib from two databases

**Figure 3:**
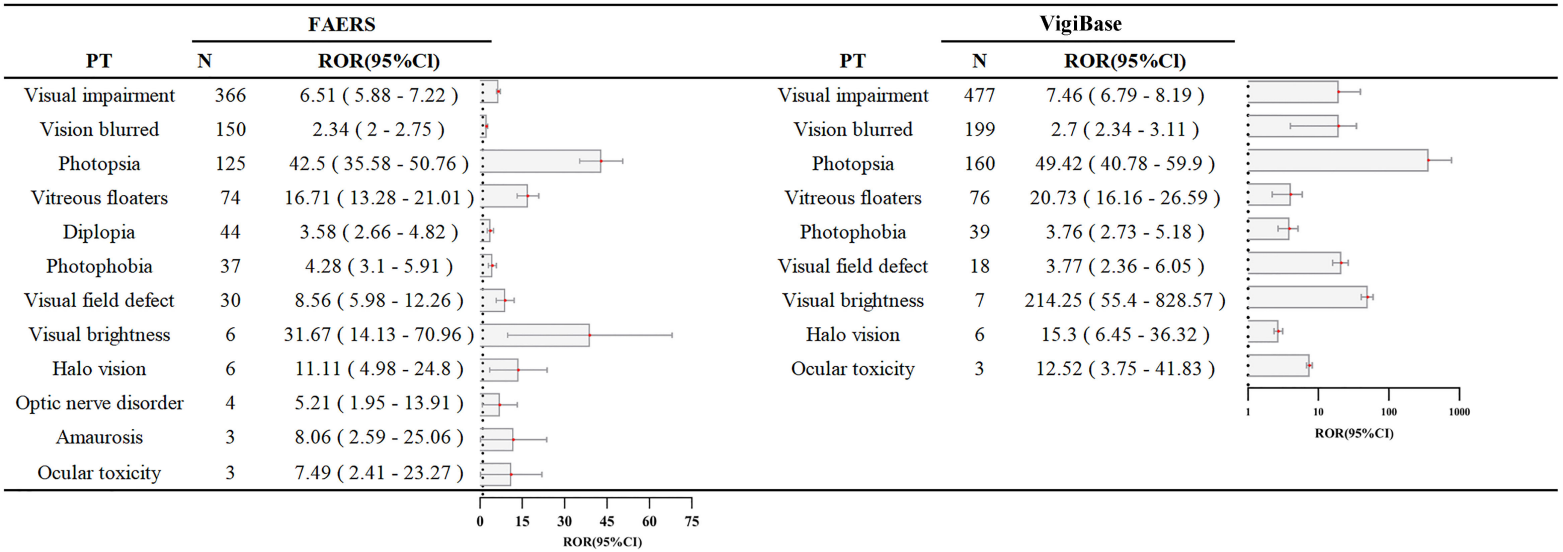
Disproportion in adverse events with crizotinib at the PT level. ROR, reporting odds ratio; CI, confidence interval. The plots show the ROR with a 95% CI on a logarithmic scale.

As shown in **Table 2**, analysis of crizotinib-related ocular AEs yielded consistent signals across both databases, with ROR of 3.46 (95% confidence interval [CI]: 3.30–3.63) in FAERS and 3.34 (95% CI: 3.17–3.51) in VigiBase, indicating a significant association between crizotinib and ocular AEs. To further characterize the spectrum of ocular toxicity, a PT-level analysis was conducted using data from both sources (**Table 3**, **Figure 2**, **Figure 3**). In FAERS, the most frequently reported PTs were visual impairment (n = 366), vision blurred (n = 150), and photopsia (n = 125). Similarly, in VigiBase, the leading PTs included visual impairment (n = 477), vision blurred (n = 199), and photopsia (n = 160), suggesting a high degree of concordance between the two databases.

**Table 3:** Intensity positive signal of crizotinib ADE from two databases.

Crizotinib	The report number	ROR (95%CI)	PRR(χ^2^)	EBGM (EBGM05)	IC (IC025)
FAERS					
Visual impairment	366	6.51 (5.88-7.22)	6.44 (1681.06)	6.43 (5.89)	2.68 (2.53)
Vision blurred	150	2.34 (2-2.75)	2.34 (114.94)	2.34 (2.04)	1.22 (0.99)
Photopsia	125	42.5 (35.58-50.76)	42.31 (4932.68)	41.41 (35.69)	5.37 (5.11)
Vitreous floaters	74	16.71 (13.28-21.01)	16.66 (1080.29)	16.53 (13.64)	4.05 (3.71)
Diplopia	44	3.58 (2.66-4.82)	3.58 (81.64)	3.57 (2.79)	1.84 (1.41)
Photophobia	37	4.28 (3.1-5.91)	4.28 (92.77)	4.27 (3.26)	2.09 (1.63)
Visual field defect	30	8.56 (5.98-12.26)	8.55 (199.24)	8.52 (6.31)	3.09 (2.57)
Visual brightness	6	31.67 (14.13-70.96)	31.66 (175.23)	31.16 (15.86)	4.96 (3.86)
Halo vision	6	11.11 (4.98-24.8)	11.11 (54.88)	11.05 (5.65)	3.47 (2.37)
Optic nerve disorder	4	5.21 (1.95-13.91)	5.21 (13.58)	5.2 (2.29)	2.38 (1.09)
Amaurosis	3	8.06 (2.59-25.06)	8.06 (18.48)	8.03 (3.11)	3.01 (1.56)
Ocular toxicity	3	7.49 (2.41-23.27)	7.49 (16.8)	7.46 (2.89)	2.9 (1.45)
VigiBase					
Visual impairment	477	7.46 (6.79-8.19)	7.41 (2449.21)	6.93 (6.41)	2.79 (2.66)
Vision blurred	199	2.7 (2.34-3.11)	2.69 (205.72)	2.64 (2.35)	1.4 (1.19)
Photopsia	160	49.42 (40.78-59.9)	49.29 (4926.1)	32.42 (27.6)	5.02 (4.75)
Vitreous floaters	76	20.73 (16.16-26.59)	20.71 (1163.07)	17.08 (13.87)	4.09 (3.74)
Photophobia	39	3.76 (2.73-5.18)	3.76 (75.96)	3.65 (2.79)	1.87 (1.4)
Visual field defect	18	3.77 (2.36-6.05)	3.77 (35.24)	3.66 (2.47)	1.87 (1.2)
Visual brightness	7	214.25 (55.4-828.57)	214.23 (445.68)	64.97 (20.95)	6.02 (4.68)
Halo vision	6	15.3 (6.45-36.32)	15.3 (68.75)	13.26 (6.43)	3.73 (2.55)
Ocular toxicity	3	12.52 (3.75-41.83)	12.52 (27.98)	11.14 (4.06)	3.48 (1.93)

PRR, the proportional reporting ratio; ROR, the reporting odds ratio; IC, the information component; EBGM, the empirical Bayes geometric mean; CI, confidence interval; 95%CI, two-sided for ROR, **χ**^2^, chi-squared; IC025 and EBGM05 lower one-sided for IC, and EBGM.

Disproportionality analysis identified strong safety signals for multiple ocular AEs associated with crizotinib, as summarized in **Table 3**. In the FAERS database, the highest ROR values were observed for photopsia (ROR = 42.5; 95% CI: 35.58–50.76), vitreous floaters (ROR = 16.71; 95% CI: 13.28–21.01), and visual brightness (ROR = 31.67; 95% CI: 14.13–70.96), indicating robust associations. Similarly, in VigiBase, photopsia (ROR = 49.42; 95% CI: 40.78–59.9) and visual brightness (ROR = 214.25; 95% CI: 55.4–828.57) demonstrated markedly elevated signal values. These results reinforce the importance of active monitoring for ocular toxicity in patients receiving crizotinib, particularly for symptoms such as visual impairment, blurred vision, and photopsia.

### 3.3 Time-to-onset analysis

**Figure 4:**
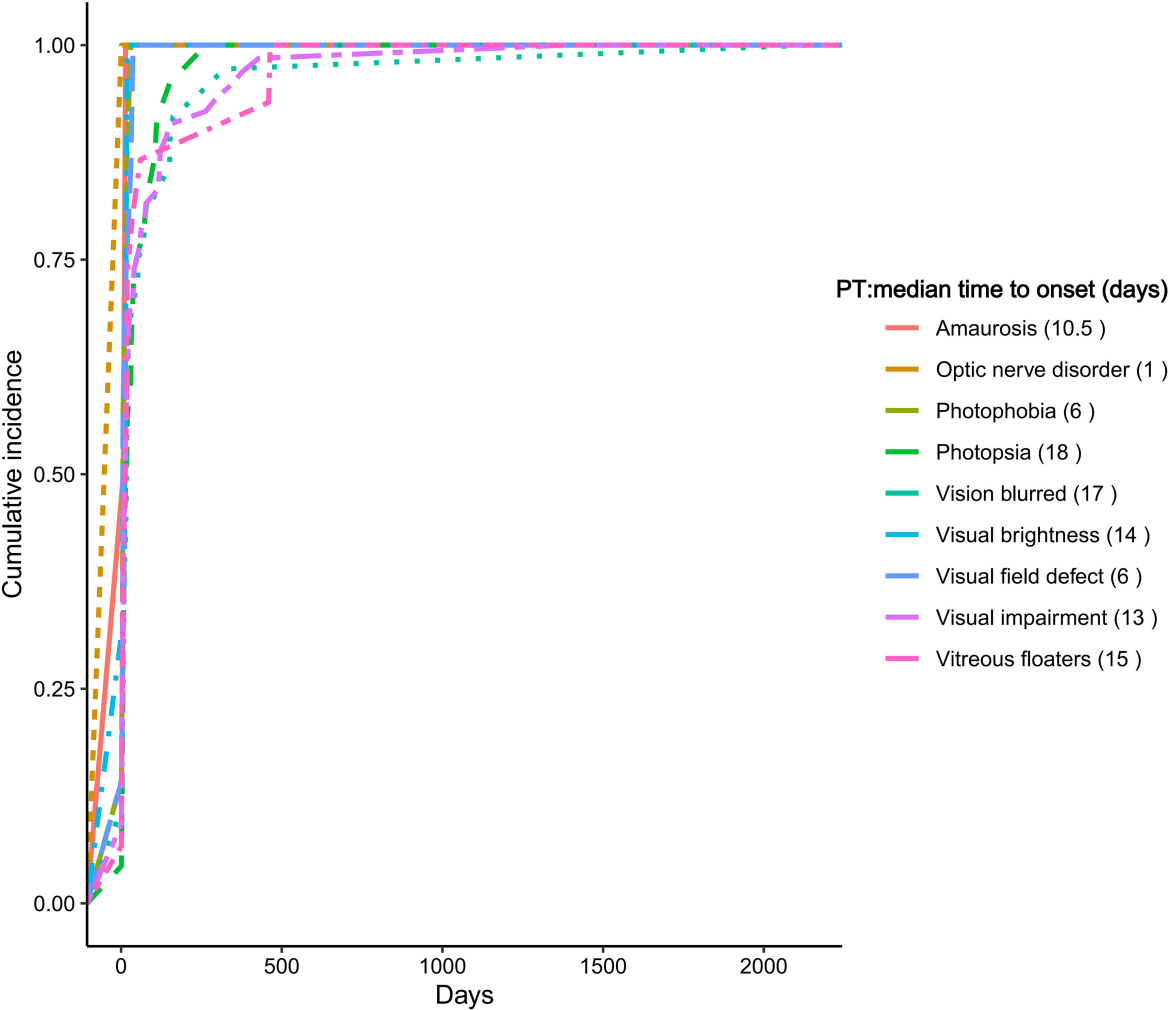
Time to onset of ocular adverse events of crizotinib.

We conducted a time-to-onset (TTO) analysis for ocular AEs associated with crizotinib using the FAERS database. Median onset times varied across PTs, with optic nerve disorder presenting the shortest median time to onset at 1 day, followed by photophobia and visual field defect at 6 days. Photopsia exhibited the longest median onset time among the commonly reported PTs, with a median of 18 days. Other notable PTs included amaurosis and vitreous floaters (both 15 days), visual brightness (14 days), visual impairment (13 days), and vision blurred (17 days). These findings underscore the early manifestation of most ocular AEs following crizotinib initiation. A detailed overview of TTO distributions is provided in **Figure 4**.

## 4 Discussion

To date, pharmacovigilance studies specifically addressing ocular AEs associated with crizotinib remain limited. This study presents, to our knowledge, the first comprehensive real-world evaluation of ocular toxicity linked to crizotinib using data from both the FAERS and VigiBase databases. Through disproportionality analysis across two independent international reporting systems, we identified consistent and robust safety signals for several ocular PTs, including visual impairment, vision blurred, and photopsia. TTO analysis further indicated that these AEs typically emerge early in the treatment course, highlighting the importance of prompt clinical monitoring.

Visual disturbances were observed in approximately 62% of patients in early clinical trials of crizotinib (17), with most events classified as low-grade and considered by investigators to have limited clinical significance (18). However, these ocular effects have not been systematically assessed in post-marketing surveillance. The present study addresses this gap by quantifying the association between crizotinib and ocular AEs using data from two large-scale pharmacovigilance databases. Our findings underscore the need to incorporate routine ophthalmologic monitoring into the clinical management of patients receiving crizotinib, particularly during the initial phase of treatment when such events are most likely to occur.

As shown in **Table 1**, ocular AEs associated with crizotinib were more frequently reported in female patients (49.19% in FAERS; 49.62% in VigiBase) than in male patients (40.21% in FAERS; 40.60% in VigiBase). This sex disparity may be attributed to the higher prevalence of ALK gene rearrangements among non-smoking females with NSCLC (19), leading to greater exposure to crizotinib in this population. Additionally, a year-by-year decline in crizotinib-related AE reports was observed from 2014 to 2025, which likely reflects the increasing clinical adoption of newer-generation ALK inhibitors with superior efficacy and safety profiles.

Our analysis of the FAERS and VigiBase databases confirms a strong association between crizotinib and a range of visual disturbances, including blurred vision, diplopia, photophobia, and vitreous floaters. In addition, robust disproportionality signals were observed for less frequently reported terms such as halo vision, amaurosis, and visual field defects. Prior clinical studies have consistently described these ocular AEs as low grade (Grade 1–2) and self-limiting (20). For instance, pivotal trials reported that approximately 60% of patients experienced vision-related symptoms, yet none required dose adjustments due to these effects (21). However, even transient visual disturbances can interfere with safety-critical activities. Expert recommendations caution that symptoms such as photopsia or progressive floaters may signal serious retinal pathology and advise patients to exercise caution when performing tasks like driving or operating machinery (20, 22).

The visual disturbances associated with crizotinib are likely attributable to its effects on retinal signal transduction pathways. In addition to ALK inhibition, crizotinib targets c-MET and ROS1 kinases (23). While ALK protein is expressed in the retinal ganglion and inner nuclear layers, c-MET appears absent from the neural retina. Experimental data have shown that crizotinib alters retinal electrophysiology—specifically, reducing the electroretinogram b-wave amplitude during dark adaptation—an effect not observed with ALK-selective inhibitors (22). Studies further demonstrated that crizotinib more potently disrupted ON/OFF ganglion cell responses compared to alectinib, indicating broader retinal functional impairment (24). These findings suggest that crizotinib’s off-target inhibition of MET and ROS1, which are expressed in retinal tissue, contributes to its ocular toxicity. By disrupting photoreceptor–bipolar cell transmission and ganglion cell signaling, crizotinib may induce characteristic symptoms such as photopsia, flashes, and blurred vision (25). Although uncommon, inflammatory or vascular complications—including optic disc edema with vasculitic leakage—have also been reported, resolving after switching to alternative ALK inhibitors (26). Collectively, these data support a biologically plausible mechanism for crizotinib-induced visual AEs, driven by interference with retinal kinase signaling pathways.

Based on these findings, we recommend incorporating proactive ophthalmologic monitoring into clinical protocols for patients receiving crizotinib. A comprehensive baseline ophthalmic examination should be conducted prior to treatment initiation to document pre-existing conditions and establish a reference point (27). Follow-up evaluations, ideally within the first month of therapy, are advised to detect early-onset visual symptoms. Throughout the treatment course, clinicians should routinely inquire about visual disturbances and document any reported symptoms. Consistent with regulatory guidance, patients should receive regular visual assessments—monthly when feasible—and be instructed to promptly report any new or worsening visual changes. Symptoms such as blurred or double vision, photopsia, halos, or visual field defects should trigger timely referral to an ophthalmologist or neuro-ophthalmologist. Unexplained visual loss or severe ocular events warrant urgent specialist evaluation. Patient education is also critical: individuals should be informed of the potential for mild visual AEs and advised to exercise caution when engaging in visually demanding activities (e.g., driving or operating machinery) until symptoms are stable (28). In clinical practice, this includes preemptive discussions about visual risks and clear guidance on activity restrictions should significant symptoms occur.

Our findings are consistent with, and expand upon, prior observations from clinical trials and small cohort studies. Visual disturbances associated with crizotinib were well documented in early-phase trials, with approximately 60–62% of patients reporting mild symptoms, typically within the first week of treatment (21). In these controlled settings, the visual effects were generally considered trivial and rarely led to treatment modification. Our large-scale pharmacovigilance analysis corroborates that most ocular AEs are indeed low-grade; however, it also reveals specific patterns—such as elevated reporting odds for photopsia, vitreous floaters, and visual brightness—that may be underrecognized in clinical trials. Prospective ophthalmologic substudies have similarly reported frequent objective findings (e.g., changes in refraction and retinal thickness on optical coherence tomography) in crizotinib-treated patients, though without serious clinical sequelae (29). Notably, Solomon et al. found that such objective abnormalities often did not correlate with patient-reported symptoms, suggesting that many visual effects of crizotinib may be subtle or subclinical (29). Our study complements these limited-sample investigations by leveraging global real-world pharmacovigilance data. By aggregating reports across diverse populations, indications, and extended treatment durations, FAERS and VigiBase capture rare or delayed events—such as optic nerve disorders—that smaller trials are likely to overlook.

Nonetheless, the intrinsic limitations of spontaneous reporting systems must be considered when interpreting these findings. Neither FAERS nor VigiBase contains denominator data (i.e., the total number of patients exposed), precluding calculation of true incidence or risk estimates (30). Underreporting is a well-recognized issue, particularly for transient, low-grade events that may not prompt medical attention. Conversely, reporting bias may inflate associations, as known or anticipated AEs are more likely to be reported. Additionally, confounding by co-medications or comorbid conditions may obscure causality. Disproportionality analysis is designed to detect statistical signals but does not establish a causal relationship between a drug and an AEs. Furthermore, both databases lack granular clinical details, such as precise onset timing or ophthalmologic examination findings, limiting mechanistic interpretation (31). Despite these limitations, the use of two independent databases enhances robustness and mitigates source-specific biases. In summary, while spontaneous reporting cannot substitute for prospective studies, our findings offer complementary real-world evidence: crizotinib-associated ocular AEs are generally mild, occur early in therapy, and follow recognizable patterns that support current monitoring practices. Future prospective studies or registries with systematic ophthalmologic assessment are warranted to clarify incidence, mechanisms, and long-term outcomes.

## 5 Conclusion

This pharmacovigilance analysis of FAERS and VigiBase revealed consistent disproportionality signals linking crizotinib to multiple ocular AEs. Strong associations were identified for blurred vision, diplopia, photophobia, vitreous floaters, halo vision, amaurosis, and visual field defects. These events typically occurred early in treatment and, while predominantly low-grade, may transiently impair visual function, particularly in tasks requiring high acuity. Notably, some events such as halo vision and amaurosis, infrequently reported in trials, emerged as significant signals in real-world data. Despite the inherent limitations of spontaneous reporting systems—such as reporting bias and absence of incidence denominators—these findings underscore the need for heightened clinical vigilance. Further prospective studies and mechanistic investigations are warranted to validate these associations and clarify the pathophysiological basis of crizotinib-induced ocular toxicity.

## Data Availability

The original contributions presented in the study are included in the article/**Supplementary Material**. Further inquiries can be directed to the corresponding author.
